# Savannah Correspondent

**Published:** 1871-04

**Authors:** 


### SAVANNAH CORRESPONDENT.

"*Drs. Powell & Goldsmith.—Gentlemen:*—I remit two dollars, which I send for the COMPANION. Please have it sent to my address. I believe in encouraging our home journals, and if you will place yours upon a high platform, will do all I can for it."

The above extract we take from a private letter, from a distinguished and large-hearted professional brother of Savannah, Ga. He is a noble representative of a local medical community, where principle asserts its power and truth exhalts the profession. We would rejoice could we see the same fraternal feeling, the same rigid adhesion to principle, the same earnestness manifested to preserve the honor and uphold the dignity of the profession, in every other portion of the State. Savannah is a bright spot upon the professional map—an oasis in the desert of professional degeneracy. It does our heart good to contemplate it; to feel that its professional men, harmonious and honorable, work by the plummet and the square; plumb their actions by the law, and square them by the ethics.

Does our brother doubt our fealty to the profession and its law? We know he does not! For this we shall labor and toil; for this, write and exhort. We have spoken—it may have been feebly and poorly—still, we have spoken. We have "set our mark" upon the errors and evils at war against the success and honor of the profession. We have, and will occupy a "high platform." Our late numbers bear us witness. We, therefore, claim that our journal is entitled to all our friend "can do for it." We expect him to do a great deal for it, as he has given us the right to so command his influence in its behalf.

---

CANNABIS INDICA IN SENILE CATARRH.—Dr. J. Curran Waring writes to us to say that he has found cannabis an invaluable remedy in catarrhus senilis. He administers it in ten minim doses gradually increased. Its effects, he says, must be seen to be thoroughly realized. He believes that as an anodyne it is immensely superior to any other drug.—*Boston Med. and Surgical*

PRESCRIPTION FOR DYSPEPSIA.—

℞—Sub. nit. bismuth, . . . . ʒ i.

Calc. Carb. Precip. . . . ʒ iii.

Fl'd. ext. gentian, . . . . ℥ ss.

Aq. Cinnamon, . . . . ℥ iijss. M.

*Salivation of Pregnancy.*—

℞—Aluminatis Sulphatis . . . ʒ iss.

Magnesiæ Sulphatis, . . . ʒ iij.

Acidi sulphuric dilut. . . . ʒ iii.

Tr. opii, . . . ʒ ss.

Mist. formyli. concen. ad . . ℥ vi. m.

S. A desert spoonful, three times daily, after eating, in a wineglasful of water.

To mist. formyli concent. is made thus :

℞—Chloroform, . . . ʒ i.

Agitate briskly, with a pint of water, until the globules of chloroform entirely disappear.

*Myalgia—Pain of Muscles.*—Dr. Austie says, "I give it in doses of ten to twenty grains, and can say that not even quinine in ague is more reliable." He also employs it in the intercostal neuralgia of sucking women.

